# Complexity of the Genetics and Clinical Presentation of Spinocerebellar Ataxia 17

**DOI:** 10.3389/fncel.2018.00429

**Published:** 2018-11-23

**Authors:** Suran Nethisinghe, Wei N. Lim, Heather Ging, Anna Zeitlberger, Rosella Abeti, Sally Pemble, Mary G. Sweeney, Robyn Labrum, Charisse Cervera, Henry Houlden, Elisabeth Rosser, Patricia Limousin, Angus Kennedy, Michael P. Lunn, Kailash P. Bhatia, Nicholas W. Wood, John Hardy, James M. Polke, Liana Veneziano, Alfredo Brusco, Mary B. Davis, Paola Giunti

**Affiliations:** ^1^Ataxia Centre, Department of Clinical and Movement Neurosciences, UCL Queen Square Institute of Neurology, London, United Kingdom; ^2^Neurogenetics Unit, National Hospital for Neurology and Neurosurgery, London, United Kingdom; ^3^Department of Neuromuscular Diseases, UCL Queen Square Institute of Neurology, London, United Kingdom; ^4^MRC Centre for Neuromuscular Diseases, UCL Queen Square Institute of Neurology, London, United Kingdom; ^5^Department of Clinical Genetics, Great Ormond Street Hospital for Children NHS Foundation Trust, London, United Kingdom; ^6^Sobell Department of Motor Neuroscience and Movement Disorders, UCL Queen Square Institute of Neurology, London, United Kingdom; ^7^Chelsea and Westminster Hospital, London, United Kingdom; ^8^Department of Neuroimmunology, UCL Queen Square Institute of Neurology, London, United Kingdom; ^9^Department of Clinical and Movement Neurosciences, UCL Queen Square Institute of Neurology, London, United Kingdom; ^10^Department of Neurodegenerative Disease, UCL Queen Square Institute of Neurology, London, United Kingdom; ^11^The Reta Lila Weston Institute of Neurological Studies, UCL Queen Square Institute of Neurology, London, United Kingdom; ^12^Istituto di Farmacologia Traslazionale – National Research Council, Rome, Italy; ^13^Department of Medical Sciences, University of Turin, Turin, Italy; ^14^Medical Genetics Unit, Città della Salute e della Scienza University Hospital, Turin, Italy

**Keywords:** PolyQ, ataxia, CAG repeat expansions, SCA17, neurodegeneration, genetic counseling

## Abstract

Spinocerebellar ataxia type 17 (SCA17) is a rare autosomal dominant neurodegenerative disease caused by a CAG repeat expansion in the TATA-box binding protein gene (*TBP*). The disease has a varied age at onset and clinical presentation. It is distinct from other SCAs for its association with dementia, psychiatric symptoms, and some patients presenting with chorea. For this reason, it is also called Huntington’s disease-like 4 (HDL-4). Here we examine the distribution of SCA17 allele repeat sizes in a United Kingdom-based cohort with ataxia and find that fully penetrant pathogenic alleles are very rare (5 in 1,316 chromosomes; 0.38%). Phenotype-genotype correlation was performed on 30 individuals and the repeat structure of their *TBP* genes was examined. We found a negative linear correlation between total CAG repeat length and age at disease onset and, unlike SCA1, there was no correlation between the longest contiguous CAG tract and age at disease onset. We were unable to identify any particular phenotypic trait that segregated with particular CAG/CAA repeat tract structures or repeat lengths. One individual within the cohort was homozygous for variable penetrance range SCA17 alleles. This patient had a similar age at onset to heterozygotes with the same repeat sizes, but also presented with a rapidly progressive dementia. A pair of monozygotic twins within the cohort presented 3 years apart with the sibling with the earlier onset having a more severe phenotype with dementia and chorea in addition to the ataxia observed in their twin. This appears to be a case of variable expressivity, possibly influenced by other environmental or epigenetic factors. Finally, there was an asymptomatic father with a severely affected child with an age at onset in their twenties. Despite this, they share the same expanded allele repeat sizes and sequences, which would suggest that there is marked difference in the penetrance of this 51-repeat allele. We therefore propose that the variable penetrance range extend from 48 repeats to incorporate this allele. This study shows that there is variability in the presentation and penetrance of the SCA17 phenotype and highlights the complexity of this disorder.

## Introduction

Spinocerebellar ataxia type 17 (SCA17; OMIM #607136) is a rare autosomal dominant neurodegenerative disorder caused by a CAG repeat expansion in exon 3 of the *TBP* that ultimately results in a loss of coordination and balance. The disease presents with various clinical symptoms at a broad range of ages at onset (3–75 years) (Stevanin and Brice, 2008). SCA17 is characterized by ataxia, pyramidal and extrapyramidal signs, cognitive impairments, psychosis, and seizures as well as involuntary movements including chorea and dystonia. It is distinct from other SCAs for its association with dementia, psychiatric symptoms, and in some patients choreic movements, hence it is also named HDL-4 (Stevanin and Brice, 2008). Brain MRI shows variable atrophy of the cerebral cortex, brain stem, and cerebellum (Rolfs et al., 2003; Toyoshima et al., 2004; Brockmann et al., 2012; Origone et al., 2018).

Typically, normal alleles have 25–40 repeats, whilst pathogenic SCA17 alleles have 49 repeats or more (Toyoshima et al., 1993). The intervening alleles (41–48 repeats) may constitute an intermediate range with variable penetrance (Toyoshima and Takahashi, 2018). Symptomatic individuals have been reported with 41 and 42 CAG/CAA repeats (Nanda et al., 2007; Nolte et al., 2010; Doherty et al., 2014, 2016; Origone et al., 2018), whilst asymptomatic individuals have been reported with 43–49 CAG/CAA repeats (Nakamura et al., 2001; Zühlke C. et al., 2003; Oda et al., 2004; Zühlke et al., 2005; Mariotti et al., 2007). The largest reported CAG/CAA repeat size to date is 66 CAG/CAA repeats (Maltecca et al., 2003). The gap between normal and expanded repeat alleles is very narrow and, combined with variable penetrance, makes it difficult to define an absolute cut-off for the pathogenic CAG/CAA repeat number in SCA17. Indeed, diagnosing SCA17 patients is often hampered by non-penetrance especially when the CAG/CAA repeat number is low. Variable penetrance is also observed in Huntington’s disease where pathogenic alleles have reduced penetrance between 36 and 39 repeats (Langbehn et al., 2004), whilst full penetrance alleles have ≥40 repeats.

**FIGURE 1:**
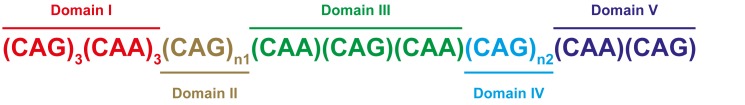
CAG/CAA repeat configuration in the *TBP* gene. SCA17 is caused by CAG repeat expansion in the TATA-box binding protein gene (*TBP*). The repeat tract is characterized by CAA interruptions and has a typical configuration as shown, and can be segmented into Domain I (red), Domain II (brown), Domain III (green), Domain IV (blue), and Domain V (purple) (Gostout et al., 1993; Gao et al., 2008). Domains II and IV are polymorphic CAG repeats.

The structure of the repeat tract in the *TBP* gene is relatively complex and can be segmented into five domains (I–V), consisting of combinations of CAG and CAA codons, which both encode glutamine leading to a pure PolyQ tract (Figure 1; Gostout et al., 1993; Gao et al., 2008). The (CAA/CAG/CAA) Domain III interruption between the polymorphic CAG repeats in Domains II and IV is present in all expanded alleles that are stably transmitted (Toyoshima et al., 1993) and its loss may lead to repeat instability (Zühlke et al., 2001, 2005; Maltecca et al., 2003). Anticipation is less common in SCA17, most likely due to the complex structure of the repeat tract and presence of CAA interruptions (Toyoshima et al., 1993; Fujigasaki et al., 2001; Rolfs et al., 2003).

Patients with 43–47 CAG/CAA repeats tend to have a parkinsonian phenotype (Kim et al., 2009; Chen et al., 2010), whilst those with 43–50 CAG/CAA repeats tend to have a phenotype resembling Huntington’s disease with psychiatric problems or dementia, parkinsonism and chorea (Stevanin et al., 2003; Bauer et al., 2004; Toyoshima et al., 2004). To date, however, there has been no association between particular repeat configurations and certain SCA17 phenotypic traits.

In this study, we examine the distribution of SCA17 allele sizes in a large United Kingdom-based cohort with ataxia and found that 38 individuals (5.78%) have pathogenic alleles in the variable or fully penetrant range (≥41 repeats), with only five patients (0.76%) in the fully penetrant (≥49 repeats) range. We selected a sub-cohort of 30 individuals with SCA17 alleles ≥41 repeats for cloning and sequencing to determine the repeat structure of their alleles. A diverse distribution of repeat configurations was observed across all cloned individuals and the CAA interruptions were present in all clones. Within our cohort we could not identify any phenotypic trait that segregated with particular CAG/CAA repeat tract structures or repeat lengths. We found that there was a negative correlation between total CAG/CAA repeat length and age at onset. Within our cohort there were three interesting sets of cases. The first was a patient who was homozygous for SCA17 alleles in the variable penetrance range. They had an age at onset similar to heterozygotes with the same repeat sizes, but also presented with a rapidly progressive dementia. In a second family there were a pair of monozygotic twins. They presented 3 years apart, with the sibling with an earlier onset having a more severe phenotype with dementia and chorea in addition to ataxia. Finally, there was an asymptomatic father with a severely affected child with an age at onset in their twenties. Despite this, they share the same expanded allele repeat sizes and sequences, which would suggest that there is marked difference in the penetrance of this 51-repeat allele. We would therefore propose that the variable penetrance range extend from 48 repeats to incorporate this allele.

## Results

### SCA17 Allele Distribution Within a United Kingdom Cohort

**FIGURE 2:**
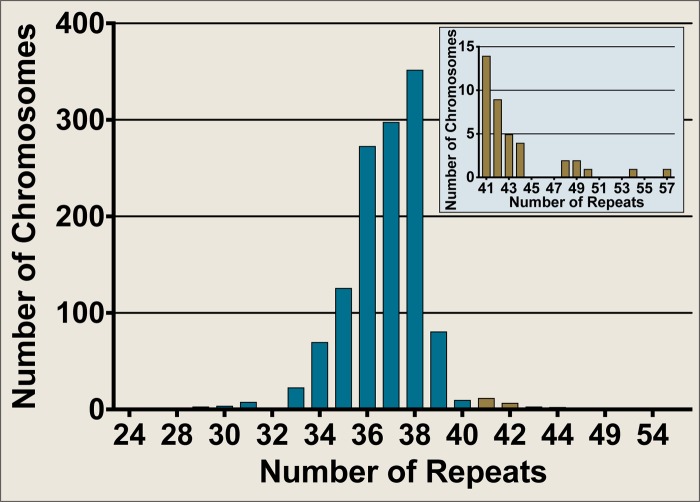
Frequency distribution of SCA17 allele sizes from diagnostic fragment analysis performed on a United Kingdom cohort at the Neurogenetics Unit, National Hospital for Neurology and Neurosurgery, London. SCA17 alleles from 1,316 discrete chromosomes were analyzed by fragment analysis. The main graph shows the frequency of all alleles tested whilst the inset depicts an enlarged view of the alleles in the variable and fully penetrant range (41–57 repeats, *n* = 39). Normal alleles (24–40 repeats, *n* = 1277) are plotted in blue, whilst variable and fully penetrant alleles are plotted in brown. The SCA17 alleles ranged from 24 to 57 repeats, with a mean allele size of 37 ± 2 repeats (mean ± standard deviation). The most frequent allele had 38 repeats (*n* = 354), whilst the most frequent variable penetrance allele had 41 repeats (*n* = 14). Only five alleles were within the fully penetrant pathogenic range (≥49 repeats).

The Neurogenetics Unit at The National Hospital for Neurology and Neurosurgery, London performed SCA17 diagnostic tests on 658 DNA samples, comprising 1,316 discrete chromosomes. The SCA17 alleles within the cohort were distributed as shown in Figure 2, with the most frequent allele having 38 repeats (*n* = 354) and 34 alleles from 33 individuals falling within the variable penetrance range between 41 and 48 repeats. An enlarged view of alleles in the variable and full penetrance range (41–57 repeats, *n* = 39) is shown in the Figure 2 insert. The most frequent variable penetrance allele had 41 repeats (*n* = 14). Only five alleles from five patients were within the fully penetrant pathogenic range – two with 49 repeats and one each with 50, 54, and 57 repeats. The average SCA17 allele size was 37 ± 2 repeats (mean ± standard deviation) and alleles ranged from 24 to 57 repeats.

### SCA17 Clone Sequence Analysis

In order to sequence the repeat regions from SCA17 alleles, a cloning strategy similar to that previously described for SCA1 was devised (Menon et al., 2013). 467 clones with 61 unique repeat region sequences were analyzed from 30 individuals with variable or fully penetrant SCA17 alleles (Supplementary Table S1). Since the number of clones sequenced for each individual differed, clone numbers were expressed as a percentage to account for clone depth (Supplementary Table S2). The most frequent allele, representing 10% of all clones, had 38 CAG/CAA repeats, whilst the most frequent variable penetrance allele represented 6.2% of all clones and had 45 CAG/CAA repeats. Two alleles of 51 and 57 CAG/CAA repeats were both the most frequent fully penetrant alleles representing 1.9% of all clones. A diverse distribution of repeat configurations was observed for all individuals and all repeat configurations contained CAA interruptions. Although it has been proposed that loss of the (CAA/CAG/CAA) Domain III interruption may lead to instability (Zühlke et al., 2001; Maltecca et al., 2003), the domain configuration 1–2–5 was only observed in 6 out of 46 (13%) of fully penetrant alleles and not at all in variable penetrant alleles. Within each individual there were different repeat configurations observed for each allele, however, the corresponding CAG/CAA repeat sizes varied by a maximum of four repeats, confirming the stability and low somatic mosaicism observed by fragment analysis (data not shown). We had SCA17 allele sequences of four patients from a cohort in Rome, Italy (Supplementary Table S3). Interestingly, two of these patients, G31 and G32, lacked the final (CAA)(CAG) Domain V interruption seen in all clones from our cohort bar one.

### Age at Disease Onset Is Negatively Correlated With Total SCA17 CAG/CAA Repeat Length

**FIGURE 3:**
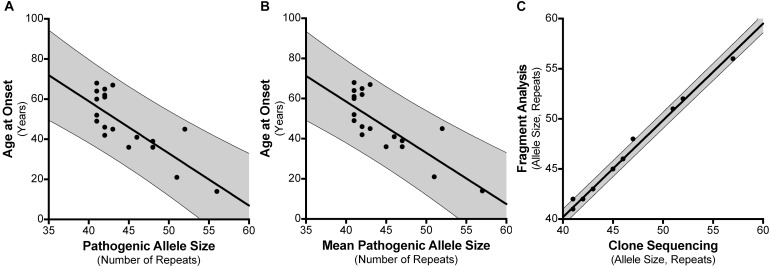
Correlation between SCA17 pathogenic allele size and age at disease onset. Age at disease onset data was available for 21 SCA17 patients that had been both analyzed by fragment sizing and clone sequencing. Pathogenic allele size as determined by fragment sizing shows a negative correlation with respect to age at onset and a good fit to the linear model (*R*^2^ = 0.595) **(A)**. Cloning and sequencing the SCA17 alleles permits the calculation of the mean pathogenic allele size, rounded to the nearest whole repeat, based on the total length of the CAG/CAA repeat tract sequence. Mean pathogenic allele size as determined by clone sequencing also shows a negative correlation with respect to age at onset and an improved fit to the linear model (*R*^2^ = 0.6005) **(B)**. Comparing the SCA17 pathogenic allele sizes determined by these two methods there is a very significant correlation between the two and a high-quality fit to the linear model (*R*^2^ = 0.9935) **(C)**. The bold line depicts the linear model fit result and the 95% confidence interval bounds are shown by the narrow line and shaded in gray.

For the 30 individuals that were cloned, age at disease onset information was available for 21 patients where there were no other confounding factors or differential diagnoses (Supplementary Table S4). For instance, individual #8 had an age at onset of 20 years which did not correspond with their SCA17 allele size of 41 repeats. Subsequent tests revealed they had a homozygous nonsense mutation (c.13642C > T; p.Gln4548^∗^; NM_182961.2) in exon 78 of the *SYNE1* gene suggesting a genetic diagnosis of ARCA1/SCAR8. We examined the influence of the repeat length on the patients’ age at onset, finding a negative correlation between the age at disease onset and the SCA17 pathogenic allele size as determined by fragment sizing (Figure 3A), with a Pearson correlation coefficient *r* = −0.7713 (significant at the 0.0001 level) and a good fit to the linear model (*A* = −2.6, *b* = 162.9, *R*^2^ = 0.595). Clone sequencing allows us to determine the mean pathogenic allele size, rounded to the nearest whole repeat, based on the total length of the CAG/CAA repeat tract sequence. This approach also displays a negative correlation between the age at onset and the mean pathogenic allele size (Figure 3B), with a Pearson correlation coefficient *r* = −0.7749 (significant at the 0.0001 level) and an improved fit to the linear model (*A* = −2.554, *b* = 160.7, *R*^2^ = 0.6005). Comparing the SCA17 allele sizing by fragment analysis and clone sequencing we observe, as expected, a very significant correlation between the two methods, with a Pearson correlation coefficient *r* = 0.9968 (significant at the 0.0001 level) and a high-quality fit to the linear model (*A* = 0.9652, *b* = 1.59, *R*^2^ = 0.9935) (Figure 3C). To examine whether the longest contiguous CAG repeat stretch was the main determinant of the age at onset, in the same way that we have previously observed for SCA1 (Menon et al., 2013), the longest CAG repeat stretch determined by clone sequencing was plotted against age at onset (Supplementary Figure S1). There was no correlation between these two variables – Pearson correlation coefficient *r* = −0.004811.

### Phenotype Distribution of Cloned SCA17 Patients

**FIGURE 4:**
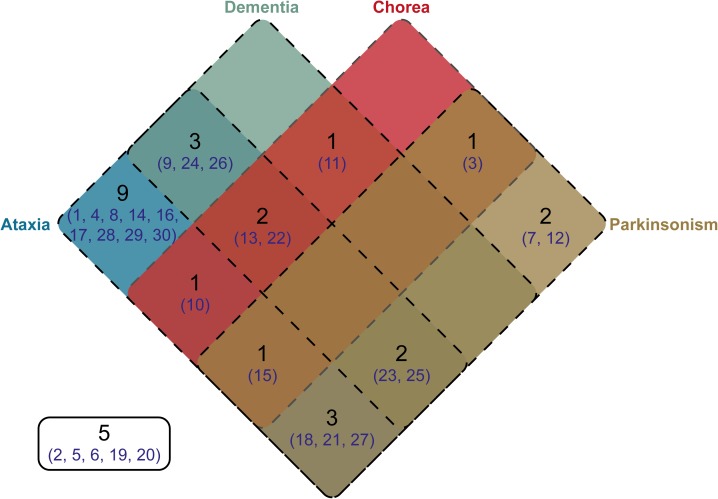
Venn diagram showing the distribution of phenotypes within the cloned SCA17 cohort. Phenotype information was available for 25 patients out of the 30 individuals in the cohort. The number of patients falling into a specific symptom category is shown in black, whilst the subject numbers corresponding to the clone frequency tables (Supplementary Tables S1, S2) are shown in dark blue. Patients with particular phenotypes are enclosed by the appropriate rounded rectangle (Ataxia, blue; Dementia, green; Chorea, red; and Parkinsonism, brown). Five subjects either were asymptomatic or no phenotype information was available.

Clinical information was available for 25 of the individuals from whom we cloned SCA17 alleles. One individual, Subject #5, was asymptomatic at the age of 50 years. Phenotypes were defined based on the presence of ataxia, dementia, chorea, and/or parkinsonism. The distribution of these phenotypes across the cohort is shown as a Venn diagram in Figure 4. As expected, the majority of subjects were ataxic (21 out of 25, 84%). Two subjects, #7 and #12, had parkinsonism only (8% of the cohort). There was no apparent segregation of particular phenotypic traits with CAG/CAA repeat tract structures or repeat lengths.

### Patient Homozygous for Variable Penetrance SCA17 Alleles

Patient #23 has SCA17 alleles of 41 and 42 CAG/CAA repeats, which both lie within the variable penetrance range. Indeed, 41 CAG/CAA repeats is the largest normal allele observed in our cohort. This individual presented with rapidly progressive cognitive impairment and balance problems at the age of 61 years old, hence there was cognitive involvement at the onset of symptoms. They underwent diagnostic tests for fragile X-associated tremor/ataxia syndrome (FXTAS) and with a Dementia Genetic Panel, but all tests were negative. MRI showed cerebellar atrophy with additional frontoparietal cortical atrophy. Their age at disease onset is consistent with heterozygotes with 41 and 42 CAG/CAA repeats within our cohort (subjects #14 and #18), in keeping with previous studies (Zühlke C.H. et al., 2003; Toyoshima et al., 2004), whilst the rapidly progressive dementia seen in our homozygote also fits with previously observed homozygotes (Zühlke C.H. et al., 2003; Toyoshima et al., 2004).

### Monozygotic Twins: Other Contributing Factors to Age at Onset or Variable Penetrance

**FIGURE 5:**
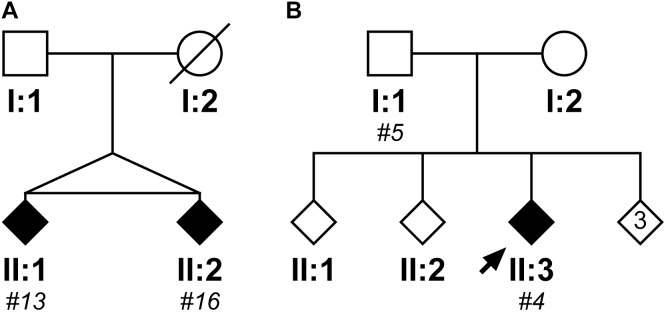
Pedigrees of two case reports from the SCA17 cloned patient cohort. The first family shows two monozygotic twins (II:1, subject #13; II:2, subject #16), who have a clone sequenced mean pathogenic allele size of 47 repeats and mean normal alleles of 38 and 37 repeats, respectively **(A)**. Subject #13 (II:1) presented with a more severe phenotype compared to their sibling, with an age of onset 3 years prior and dementia/cognitive impairment and chorea in addition to the ataxia observed in their sibling (Subject #16, II:2). The second family shows a father (I:1, subject #5) and child (II:3; subject #4) where the father was asymptomatic at the age of 50 years old, whilst the child developed ataxic symptoms at the age of 21 years old **(B)**. Despite the apparent anticipation, they both had a clone sequenced mean pathogenic allele size of 51 repeats, but their normal alleles differed with the child having a slightly larger mean allele size of 39 repeats compared to 37 repeats in their father. DNA was only available for individuals who were cloned, with the given subject number in italics.

Within the cloned cohort there were a pair of monozygotic twins, subjects #13 and #16, whose pedigree is shown in Figure 5A. They presented 3 years apart, both with memory impairment followed by weight loss. Although there were no DNA samples available from other family members to confirm SCA17, there was a history of “fidgety hands” in one parent (who died aged 45 from breast cancer) and their grandparent. Interestingly, subject #13 (II:1) not only presented 3 years earlier than their sibling, but also had a more severe phenotype with dementia/cognitive impairment and chorea in addition to the ataxia seen in their sibling. Fragment sizing analysis shows that both twins have the same pathogenic and normal allele sizes (48 and 38 repeats, respectively). Further analysis by clone sequencing revealed that both twins have the same size pathogenic allele (47 repeats) with a single repeat difference in their normal alleles (38 and 37 repeats, respectively). However, this difference could be explained by a single, potentially artefactual clone of 34 repeats skewing the mean allele size in subject #16. Indeed, the sequence of the twins’ repeat configurations were also very similar. This could be a case of variable expressivity or there may be other modulators influencing the age at disease onset and severity such as environmental or epigenetic factors.

### Father and Child: Anticipation or Variable Penetrance

Within the cloned cohort there were a father (subject #5, I:1) and child (subject #4, II:3) who displayed a classic case of clinical anticipation, whereby the father was asymptomatic at the age of 50 and the child first developed ataxic symptoms and slurred speech at the age of 21 years old. Their pedigree is shown in Figure 5B. Despite this anticipation in symptoms, there is no concomitant expansion in the number of CAG repeats during paternal transmission (the pathogenic allele contained 51 repeats both by fragment sizing and clone sequencing). Examining the clone sequences, both father and child have the same repeat configurations in their pathogenic alleles. There is, however, a difference in their normal alleles with the child having a slightly larger allele size of 39 repeats compared to 37 repeats in their father. STR analysis confirms that these are related, but not identical samples (Supplementary Table S5). Since there is no genetic basis for anticipation from intergenerational expansion of the CAG/CAA repeat, this would suggest that this is a case of variable penetrance, despite the pathogenic allele being within the typical full penetrance range.

## Discussion

This study demonstrates the complexity in the genetic and phenotypic presentation of SCA17, which poses a challenge for geneticists and clinicians alike, who encounter SCA17 families in genetic counseling. Here we present data from the largest United Kingdom-based ataxic cohort to-date that was diagnostically tested for SCA17 by the Neurogenetics Unit at The National Hospital for Neurology and Neurosurgery, London. Out of the 658 genetic tests, the most frequent allele had 38 repeats (*n* = 354), which is in keeping with previous studies (Alendar et al., 2004, Nolte et al., 2010). The most frequent pathogenic allele was in the variable penetrance range (41–48 repeats) and had 41 repeats (*n* = 14), whilst only five alleles were observed in the fully penetrant range (≥49 repeats). The distribution observed in our cohort is reflective of the data previously shown for a smaller independent cohort from the north east of England (Craig et al., 2005).

To understand whether the structure of the repeat can modulate the phenotype in SCA17 we cloned and sequenced the *TBP* gene CAG/CAA repeat tract from 30 individuals within the cohort with variable or fully penetrant alleles of at least 41 repeats. 467 clones were sequenced with 61 unique repeat region configurations. The most frequent allele sequence had 38 CAG/CAA repeats and comprised 10% of all clones generated, which is in keeping with previous studies (Zühlke et al., 2001) and fits with the greater population of the cohort analyzed by diagnostic fragment analysis. It has been suggested that loss of the (CAA)(CAG)(CAA) Domain III interruption between the polymorphic CAG repeats of Domains II and IV may lead to repeat instability (Zühlke et al., 2001; Maltecca et al., 2003). In our cohort, the compete loss of this interruption is not observed in any of the variable penetrant alleles (none of the 186 clones between 41 and 48 repeats) and only seen in 6 out of 46 (13%) of the fully penetrant allele clones. Indeed, there were only 2 clones with 40 repeats, and one clone each with 14, 25, 27, and 29 repeats lacking Domain III out of 235 normal allele clones. This would suggest that if such instability events do occur they are not incremental or stepwise, as one would expect to observe clones lacking Domain III in the variable penetrance range, however, examination of a larger cohort that includes families with multiple generations would be required to confirm this. This also suggests that such unstable alleles do not account for the variability in penetrance of the SCA17 phenotype.

We examined the influence of the SCA17 allele repeat length determined by both fragment sizing and clone sequencing on the patients’ age at disease onset for 21 of the patients. We found that there was a good negative correlation between age at onset and total repeat length, in keeping with previous studies (Toyoshima et al., 2004; Craig et al., 2005; Gao et al., 2008). The data had a good fit to a linear model, which was further improved when using mean pathogenic allele size determined from clone sequencing compared to fragment sizing (*R*^2^ = 0.6005 vs. 0.595). For SCA1, we previously suggested that the longest contiguous CAG repeat stretch was the main determinant of age at disease onset (Menon et al., 2013). To validate whether this was the case for SCA17, the mean longest CAG repeat stretch determined by clone sequencing was plotted against age at onset, however, we found no correlation between these two variables. The complexity of the repeat configuration with CAA interruptions that also encode glutamine at the protein level would support total repeat length as being a main factor in determining the age at onset. Since the CAA interruptions are not completely lost in the expanded alleles in our cloned cohort, the effect of the CAA interruption is unclear, however, the secondary structures formed may have a stabilizing influence during DNA replication and breaking up the repetitive sequence into shorter homogenous triplet tracts could reduce slippage between complementary strands (Choudhry et al., 2001; Zühlke et al., 2005).

There is controversy over whether alleles with 41 and 42 repeats can be considered pathogenic given their frequency in normal individuals (1.5 and 0.6%, respectively) (Shin et al., 2015). In our cohort, we observe alleles with 41 and 42 repeats at a similar frequency of 1.1 and 0.68%, respectively. However, we have examined a cohort selected based on their genetic tests for an ataxic phenotype or pre-symptomatic testing for such a disorder. This introduces bias that means we cannot extrapolate our findings to the general population. Although caution should be used when referring to these alleles as pathogenic given the potential for coincidental phenotypes occurring by chance, we believe that they are indeed contributing to the phenotype in our cohort since their inclusion in analysis improves the correlation between pathogenic allele size and age at disease onset and the fit to the linear model with tighter confidence interval boundaries (Supplementary Figure S2).

Our cloned cohort had a diverse distribution of symptoms when defined by the categories of ataxia, dementia, chorea, and parkinsonism. Since most of these patients were referred for SCA17 diagnostic testing based on their ataxic phenotype, it was no surprise that 84% had ataxia as a symptom with 57% of these also having another symptom be it dementia, chorea and/or parkinsonism. Interestingly, two patients (8% of the cohort) only had parkinsonism as a symptom. Previous studies have found that patients with 43–50 CAG/CAA repeats tend to have symptoms resembling Huntington’s disease (dementia, chorea, and parkinsonism) (Stevanin et al., 2003; Bauer et al., 2004; Toyoshima et al., 2004), whilst those with 43–47 CAG/CAA repeats tend to have a parkinsonian phenotype (Kim et al., 2009; Chen et al., 2010). This does not appear to be the case in our cohort with 3 out of 6 patients with chorea (subjects #3, #10, #13; 50%), 1 out of 8 patients with dementia (subject #13; 12.5%), and 3 out of 9 patients with parkinsonism (subjects #3, #7, #27; 33.3%) having 43–50 CAG/CAA repeats. There does not appear to a particular repeat structure or length that can be associated with a particular phenotype. This is probably due to several other confounding factors including the variable expression and penetrance of this condition.

Within our cloned cohort there was one patient who was homozygous for SCA17 variable penetrance alleles of 41 and 42 CAG/CAA repeats. To date there have been four homozygotes and one compound heterozygote reported (Zühlke C.H. et al., 2003; Oda et al., 2004; Toyoshima et al., 2004; Hire et al., 2011). Our patient had an age at onset that was consistent with equivalent pathogenic allele heterozygotes within our cohort, which is in keeping with previous reports (Zühlke C.H. et al., 2003; Toyoshima et al., 2004). It is interesting to note that this patient presented with severe cognitive impairment with rapid progression despite the late onset. This would suggest a contribution of the 41-repeat allele to the pathogenesis of SCA17 in this patient even though both alleles are at the lower end of the variable penetrance range.

Within our cohort there was a pair of monozygotic twins. Interestingly, they presented with symptoms 3 years apart and the sibling that presented first (subject #13) also had a more severe phenotype with dementia/cognitive impairment and chorea in addition to ataxia that their sibling had. Clone sequencing showed that they had the same mean pathogenic allele size and similar repeat configurations. There was, however, a single repeat difference in their normal alleles, which may be due to a single potentially artefactual clone of 34 repeats skewing the mean in subject #16. A striking phenotypic discordance between monozygotic twins is not uncommon and has previously been observed in twin studies for Huntington’s Disease and SCA2 (Levy et al., 1999; Anderson et al., 2002; Panas et al., 2008; Ketelaar et al., 2012). Although this could be viewed as a case of variable expressivity, it is also possible that there are other modulators influencing the age at disease onset and disease severity such as environmental or epigenetic factors.

A father and child within our cohort displayed marked anticipation, whereby the father (subject #5) was asymptomatic at the age of 50 and his child (subject #4) was severely affected at the age of 21. However, they shared the same mean pathogenic allele size with 51 repeats, which should be fully penetrant, suggesting that there was no genetic basis for the anticipation. They did, however, differ by two repeats in their normal allele with the affected child having the larger normal allele. Such a striking phenotypic difference with the same pathogenic allele would suggest that this is a case of variable penetrance, possibly influenced by other environmental or epigenetic factors. We therefore recommend that the variable penetrance range be extended from 48 repeats to incorporate this 51-repeat allele. Interestingly, subject #9 within the cohort had a 52-repeat pathogenic allele and an age at disease onset of 45 years old. This patient showed cerebellar and cerebral atrophy at this age. By the age of 59, subject #9 required a frame to walk and their cognitive function was impaired. This demonstrates the variability in the clinical presentation of SCA17 given a particular CAG/CAA repeat tract size.

## Conclusion

In conclusion, we have presented the distribution of allele sizes across normal, variably penetrant and fully penetrant ranges in the largest United Kingdom-based ataxic cohort to-date. We found that fully penetrant alleles in our cohort were very rare (5 in 1,316 chromosomes; 0.38%). Cloning and sequencing a sub-cohort of 30 individuals revealed no particular repeat structure or length that could be associated with a particular SCA17 phenotype, however, there was a good negative correlation between mean total pathogenic allele size and age at disease onset. We observed a variable phenotype in a pair of monozygotic twins, who had the same mean pathogenic allele size and repeat structures, suggesting other factors such as environmental or epigenetic factors are modulating their phenotype. Finally, we found a stark dichotomy in the phenotype of an asymptomatic father and severely affected child who share the same mean pathogenic allele size of 51 repeats and consisting of the same repeat structures. Since this mean pathogenic allele falls within the existing fully penetrant range yet displays variable penetrance, we advise that the variable penetrance range be increased from 48 repeats to include this allele.

This study has demonstrated the variability in the presentation and penetrance of the SCA17 phenotype highlighting the complexity of genetically diagnosing and counseling families with this rare condition.

## Materials and Methods

### Ethics Statement

This research has been approved by the London (Queen Square) NHS Research Ethics Committee (reference 09/H0716/53) at the National Hospital for Neurology and Neurosurgery, London.

### Patient Cohort

Blood from patients with an ataxic phenotype or pre-symptomatic family members were sent to the Neurogenetics Unit at The National Hospital for Neurology and Neurosurgery, London for SCA17 diagnostic testing. Thirty individuals were selected for cloning and sequencing based on having SCA17 expanded alleles in the variable to fully penetrant range (≥41 repeats). This cloned cohort consisted of 14 males and 16 females with ages at disease onset ranging from 14 to 68 years old. Ethnicity was predominantly British (19 individuals), whilst 5 were from the Indian subcontinent, 4 were of Spanish/Mediterranean heritage, and 2 were of unknown ethnicity. Fragment analysis and sequence information for an additional four patients were available to us from the Institute of Translational Pharmacology in Rome, Italy.

### SCA17 Fragment Sizing

Genomic DNA was extracted from patient peripheral blood leukocytes using a FlexiGene DNA kit (QIAGEN). SCA17 alleles were amplified by PCR using GoTaq DNA polymerase (Promega) and the primers SCA17For (FAM-5′-GATGCCTTATGGCACTGGACTG-3′) and SCA17Rev (5′-CTGCTGGGACGTTGACTGCTG-3′). PCR products were checked on a 4% (w/v) agarose gel and then the fragments were resolved on an ABI 3730*xl* DNA analyser with a GeneScan 500 LIZ Size Standard (Thermo Fisher Scientific). Fragment analysis was performed with GeneMapper software (version 4.0, Applied Biosystems) and the most intense peak for each allele was selected to calculate the allele size. Repeat lengths were calculated by subtracting the number of extraneous bases in the PCR product outside of the repeat region (120 bp) and then dividing by 3. A correction factor of two repeats is then added, based on the difference previously seen between fragment analyzed and sequenced clones within the laboratory (data not shown).

### Cloning of SCA17 Allele CAG/CAA-Repeat Tracts

Spinocerebellar ataxia type 17 allele CAG/CAA-repeat tracts from 30 patients from this cohort were PCR amplified using Phusion High-Fidelity DNA polymerase (New England Biolabs) and primers which flank the CAG/CAA-repeat region and introduce restriction enzyme cleavage sites. The primers used were *TBP*x3 *Bam*HI Forward (5′-ATGTTTggatccTCCACAGGGTGCCATGAC-3′) and *TBP*x3 *Xho*I Reverse (5′-GGTTTGctcgagACACGAAGTACTCACTGC-3′), with restriction sites indicated in lower case. The PCR reactions were processed and cloned into a pcDNA3.1(+) vector similar to previously described for SCA1 alleles (Menon et al., 2013) or pCR-Blunt using the Zero Blunt PCR Cloning Kit (Thermo Fisher Scientific). Clone plasmids were propagated in Stbl3 *E. coli* (Thermo Fisher Scientific) (genotype F^−^ *mcr*B *mrr hsd*S20(r_B_^−^, m_B_^−^) *rec*A13 *sup*E44 *ara*-14 *gal*K2 *lac*Y1 *pro*A2 *rps*L20(Str^R^) *xyl*-5 λ^−^ *leumtl*-1). Plasmids were sequenced using the BigDye Terminator v3.1 Cycle Sequencing Kit (Thermo Fisher Scientific).

### Sample Validation

The PowerPlex 16 HS System (Promega) was used to verify sample identity for #13 and #16 (monozygotic twins) and #5 and #4 (father and child, respectively). 0.5 and 1 ng genomic DNA was used as template for the PCR amplification according to the manufacturer’s protocol. Fragments were resolved on an ABI 3730*xl* DNA analyser (Thermo Fisher Scientific). Analysis was performed using the Applied Biosystems Microsatellite Analysis module on the Thermo Fisher Cloud.

### Statistical Analysis

Analyses were only performed on individuals with a SCA17 diagnosis where age at disease onset data were available (*n* = 21). A bivariate two-tailed Pearson correlation between the pathogenic allele repeat size and the age at disease onset was performed. In addition, the data were fit to a linear model of the form “Age at Onset = A ^∗^ (Pathogenic Allele Size) + b.” 95% confidence interval bounds are shown. All statistics were calculated in Prism (version 7.0d, GraphPad Software, Inc.).

## Author Contributions

SN, MS, RL, MD, and PG conceived and designed the experiments. SN, WL, HG, AZ, RA, SP, CC, HH, ER, PL, AK, ML, KB, NW, and LV performed the experiments and collated the patient phenotypic information. SN, WL, HG, SP, MS, RL, CC, JP, LV, AB, MD, and PG analyzed the data. SN and WL wrote the first draft of the manuscript. All authors contributed to manuscript revision.

## Conflict of Interest Statement

The authors declare that the research was conducted in the absence of any commercial or financial relationships that could be construed as a potential conflict of interest.

## Supplementary Material

FIGURE S1 The longest contiguous CAG repeat stretch in the SCA17 pathogenic allele size does not influence age at disease onset. Age at disease onset data was available for 21 SCA17 patients that had been both analyzed by fragment sizing and clone sequencing. Cloning and sequencing the SCA17 alleles allows the configuration of the repeat tract to be determined. To examine whether the longest contiguous CAG repeat stretch influenced the age at onset, similarly to what we previously observed in SCA1 (Menon et al., 2013), the mean longest CAG repeat stretch for each individual was plotted against age at onset. There was no correlation between these two variables. The bold line depicts the linear model fit result and the 95% confidence interval bounds are shown by the narrow line and shaded in gray.

FIGURE S2 Patients with 41 and 42 repeat alleles contribute to improving the correlation between SCA17 pathogenic allele size and age at disease onset. Age at disease onset data was available for 21 SCA17 patients that had been both analyzed by fragment sizing and clone sequencing and this data is plotted in red. If patients with 41 and 42 repeats are excluded from the analysis, only 9 patients remain, plotted in green. The bold line depicts the linear model fit result and the 95% confidence interval bounds are shown by the narrow line and shaded area. It is clear that the inclusion of the patients with 41 and 42 repeats improves the negative Pearson correlation (*r*) and the fit to the linear model (*R*^2^) with tighter confidence interval boundaries.

TABLE S1 Frequency with which each clone sequence was detected for each individual in our cohort. The bold lines indicate the variable penetrance range between 41 and 48 repeats. Domains 1–5 refer to the Domains I–V of the repeat structure as depicted in Figure 1. 467 clones with 61 unique repeat region sequences were analyzed from 30 individuals with variable of fully penetrant SCA17 alleles.

TABLE S2 Frequency with which each clone sequence was detected for each individual in our cohort, corrected for the clone depth sequenced for each patient. The bold lines indicate the variable penetrance range between 41 and 48 repeats. Domains 1–5 refer to the Domains I–V of the repeat structure as depicted in Figure 1. 467 clones with 61 unique repeat region sequences were analyzed from 30 individuals with variable of fully penetrant SCA17 alleles. The most frequent allele had 38 CAG/CAA repeats and was found in 47/467 clones (10%). All repeat configurations contained CAA interruptions.

TABLE S3 Fragment and sequence analysis of SCA17 alleles in four patients in an Italian cohort. SCA17 alleles were sized by fragment analysis and subsequently sequenced at the Institute of Translational Pharmacology, Rome. Interestingly, two of these patients, G31 and G32, lack the final (CAA/CAG) Domain V interruption which is observed in all clones from the United Kingdom cohort bar one. These patients also had a later age at disease onset for their expanded allele size, compared to patients with similar sized alleles (#4, 51 repeats, 21 years; #9, 52 repeats, 45 years).

TABLE S4 Summary of age at disease onset and allele size data for each individual. Data is presented from both fragment sizing and clone sequencing normal and expanded alleles. Age at onset data was available for 21 out of 30 individuals. Patient #8 (highlighted in blue italics) had a genetic diagnosis of ARCA1/SCAR8 (see text), which explains the early age at onset despite the 41 repeat SCA17 allele. This data was used to compile the graphs in Figure 3.

TABLE S5 Short tandem repeat (STR) analysis to confirm identity of individuals #4, 5, 13, and 16. The PowerPlex 16HS System was used to amplify microsatellite markers at 15 STR loci. Numbers represent the alleles (repeat numbers) at that locus. This data confirms that father (#5) and child (#4) are separate samples and yet have the same 51-repeat pathogenic alleles with marked difference in phenotype. The monozygotic twins (#13 and #16) share the same alleles across all loci tested.

## Abbreviations

CAA: cytosine, adenine, adenine
CAG: cytosine, adenine, guanine
HDL-4: Huntington’s disease-like 4
MRI: magnetic resonance imaging
PCR: polymerase chain reaction
PolyQ: polyglutamine
SCA: spinocerebellar ataxia
STR: short tandem repeat
TBP: TATA-box binding protein.
