# Supplementary figures and images for: Complexity of the Genetics and Clinical Presentation of Spinocerebellar Ataxia 17
Source: Front Cell Neurosci. 2018 Nov 23;12:429. doi: 10.3389/fncel.2018.00429 (PMC6265347; doi:10.3389/fncel.2018.00429)

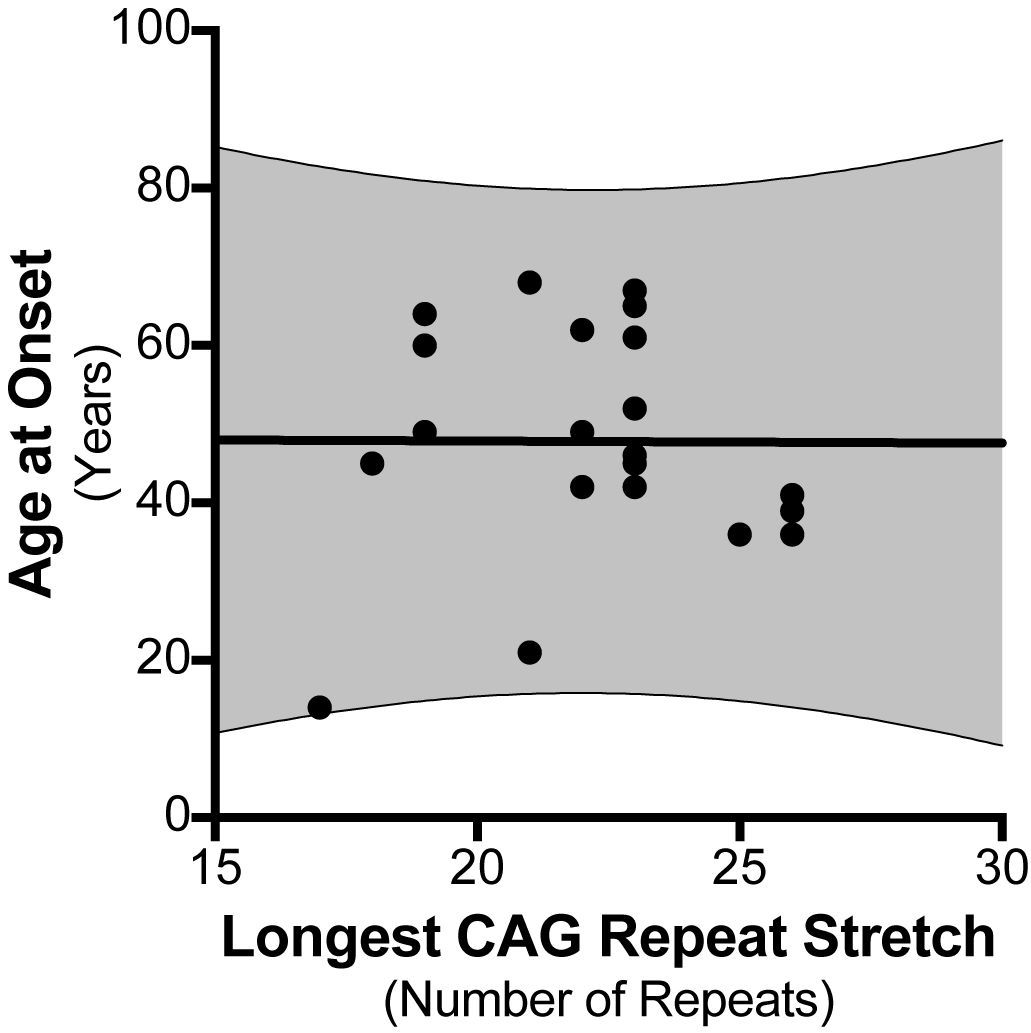

Supplement: FIGURE S1 — The longest contiguous CAG repeat stretch in the SCA17 pathogenic allele size does not influence age at disease onset. Age at disease onset data was available for 21 SCA17 patients that had been both analyzed by fragment sizing and clone sequencing. Cloning and sequencing the SCA17 alleles allows the configuration of the repeat tract to be determined. To examine whether the longest contiguous CAG repeat stretch influenced the age at onset, similarly to what we previously observed in SCA1 (Menon et al., 2013), the mean longest CAG repeat stretch for each individual was plotted against age at onset. There was no correlation between these two variables. The bold line depicts the linear model fit result and the 95% confidence interval bounds are shown by the narrow line and shaded in gray. [file Image_1.TIF]

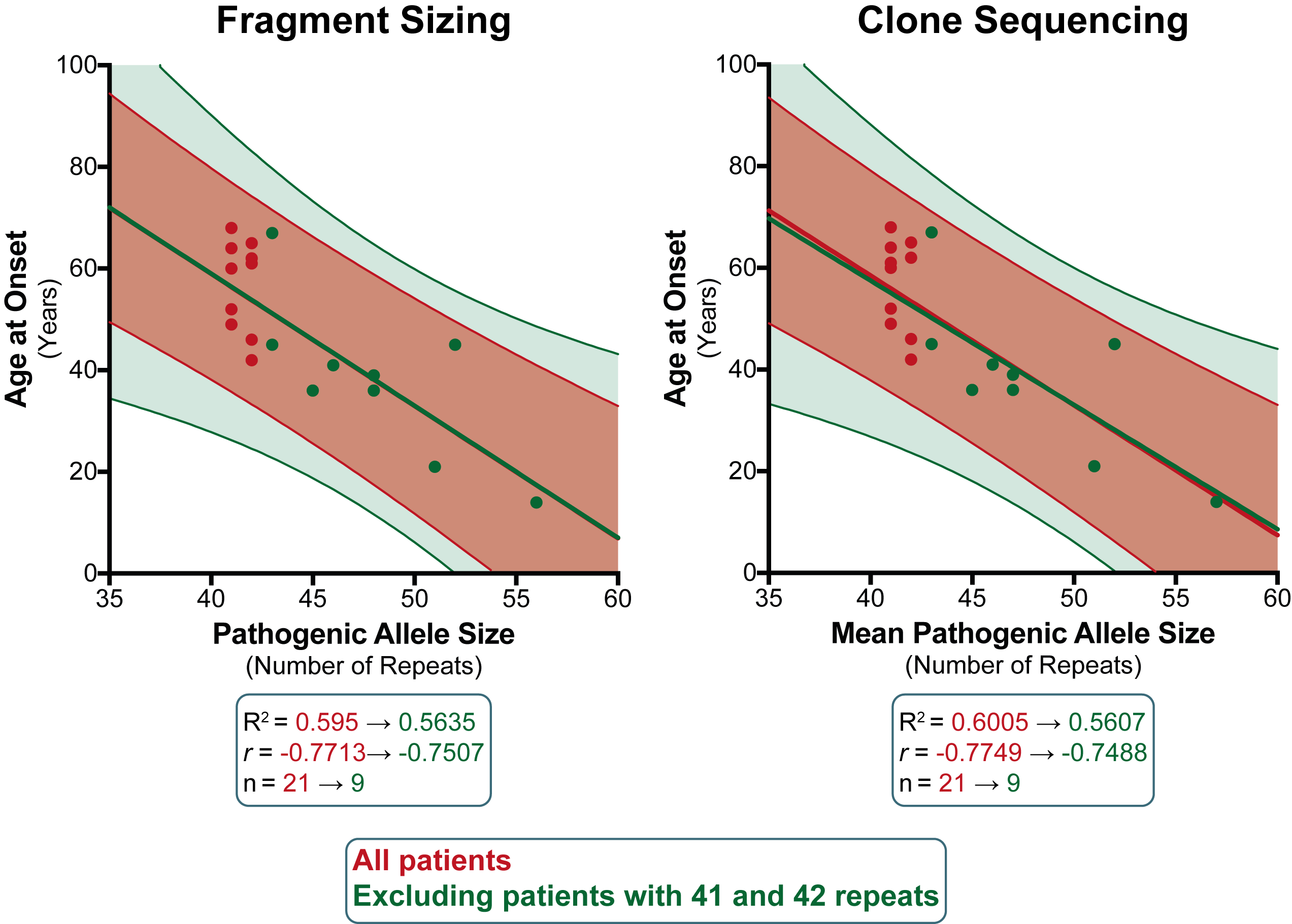

Supplement: FIGURE S2 — Patients with 41 and 42 repeat alleles contribute to improving the correlation between SCA17 pathogenic allele size and age at disease onset. Age at disease onset data was available for 21 SCA17 patients that had been both analyzed by fragment sizing and clone sequencing and this data is plotted in red. If patients with 41 and 42 repeats are excluded from the analysis, only 9 patients remain, plotted in green. The bold line depicts the linear model fit result and the 95% confidence interval bounds are shown by the narrow line and shaded area. It is clear that the inclusion of the patients with 41 and 42 repeats improves the negative Pearson correlation (r) and the fit to the linear model (R2) with tighter confidence interval boundaries. [file Image_2.TIF]
